# Optimization of protein removal process of *Lonicera japonica* polysaccharide and its immunomodulatory mechanism in cyclophosphamide‐induced mice by metabolomics and network pharmacology

**DOI:** 10.1002/fsn3.3067

**Published:** 2022-09-25

**Authors:** Jie Ding, Haitao Du, Haining Tan, Jing Li, Lingna Wang, Li Li, Yongqing Zhang, Yuhong Liu

**Affiliations:** ^1^ School of Pharmaceutical Sciences Shandong University of Traditional Chinese Medicine Jinan China; ^2^ Shandong Key Laboratory of Carbohydrate Chemistry and Glycobiology, National Glycoengineering Research Center Shandong University Qingdao China; ^3^ Sishui Siheyuan Culture and Tourism Development Company, Ltd Shandong China

**Keywords:** *Lonicera japonica* polysaccharide, metabolomics, network pharmacology, protein removal

## Abstract

In this study, TCA–n‐butanol was chosen as the best deproteinization method for *Lonicera japonica* polysaccharide (LJP) by comparing the polysaccharide retention rate and the protein clearance rate of five different methods. The response surface methodology (RSM) based on the Box–Behnken design (BBD) was used to optimize the deproteinization conditions as follows: TCA: n‐butanol = 1: 5.1, polysaccharide solution: (TCA–n‐butanol) = 1: 2.8, and shook for 33 min. LJP could promote the thymus and spleen indexes of cyclophosphamide (CTX)‐induced immune‐deficient mice. Besides, the contents of cytokine interleukin‐2 (IL‐2) and hemolysin in mice serum were augmented after treatment with LJP. Based on serum metabolomics analysis, a total of 14 metabolites (VIP >1.0, FC >2 or FC <0.5, and *p* value < .05) were selected as the potential biological biomarkers related to the LJP for treating CTX‐induced mice. After the pathway enrichment analysis, these metabolites were mainly involved in the relevant pathways of arginine biosynthesis, Citrate cycle, and other metabolic pathways. Network pharmacology further showed that there were 57 key targeting proteins in the intersection of the potential biological biomarkers and immunodeficiency‐related targeting proteins according to protein–protein interactions analysis (PPI). The biological function analysis indicated that the potential biological processes were mainly associated with tricarboxylic acid (TCA) cycle, phospholipid metabolic process, metabolic process, and so on. In conclusion, serum metabolomics combined with network pharmacology could be helpful to clarify the immunomodulatory mechanism of LJP and provide a literature basis for further clinical research on the therapeutic mechanism of LJP.

## Graphical abstract

In this paper, the best protein removal method of *Lonicera japonica* polysaccharide was determined and its process was optimized by response surface method. Pharmacological experiments showed that polysaccharides had therapeutic effect on immunodeficiency mice induced by cyclophosphamide (CTX). The immunomodulatory mechanism of polysaccharide was elucidated by the combination of serum metabolomics and network pharmacology.

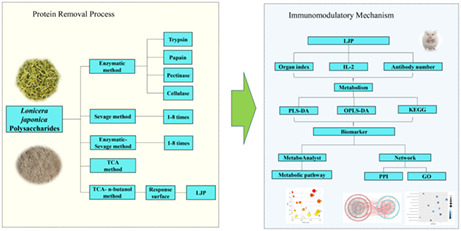

## 1 INTRODUCTION

*Lonicera japonica*, also named “Jin Yin Hua” or “Ren Dong” in China, is a well‐known traditional Chinese medicine (TCM) to treat fever, arthritis, respiratory infection, and epidemic disease (Liu, Fang, et al., 2018; Wang et al., 2017). Nowadays, some evidence indicate that *Lonicera japonica* might have a significant effect on COVID−19 patients by reducing fever and improving breathing (Zhao et al., 2021). Recent pharmacological studies have shown that *Lonicera japonica* polysaccharides have a broad spectrum of biological activity, including anti‐allergic, antipancreatic cancer, hypoglycemic, hypolipidemic, and neuroprotective effects (Lin et al., 2016; Liu, Huang, & Hu, 2018; Tian et al., 2012; Wang et al., 2017; Zhao et al., 2018). However, we found that polysaccharides extracted by water often contain more protein, which affects further study of their chemical properties and pharmacological activities (Cao et al., 2022; Younes & Rinaudo, 2015). To optimize the best approach and the best condition of deproteinization, five methods were investigated, including the enzymatic method, Sevage method, enzymatic–Sevage method, trichloroacetic acid (TCA) method, and TCA–n‐butanol method. Response surface methodology (RSM) was used to optimize protein removal conditions.

In recent years, the immunomodulatory activities of polysaccharides from TCM were one of the most critical biological activities. Although relevant literature reports show that *Lonicera japonica* polysaccharides had immunomodulatory functions, the mechanism of action is still not fully understood (Zhou et al., 2018). Metabolomics is a new biological technology that provides a powerful method to study the biomarkers, the changes in biological systems, and is associated with physiological and pathophysiological processes. This method provides comprehensive qualitative and quantitative analysis of metabolites in vivo, providing vital information on chemical biomarkers, and could be used for determining taxonomy and quality control. Therefore, metabolomics could better demonstrate the key metabolites, pathways, and main regulatory processes of immunocompromised mice. Network pharmacology could explain the synergy of TCM with compounds and multiple target substances, thereby systematically revealing the pathogenesis and molecular understanding of drugs. Metabolomics combined with network pharmacology could clarify the mechanism and target of TCM at the level of systemic biology and whole integration (Ren et al., 2020).

**FIGURE 1:**
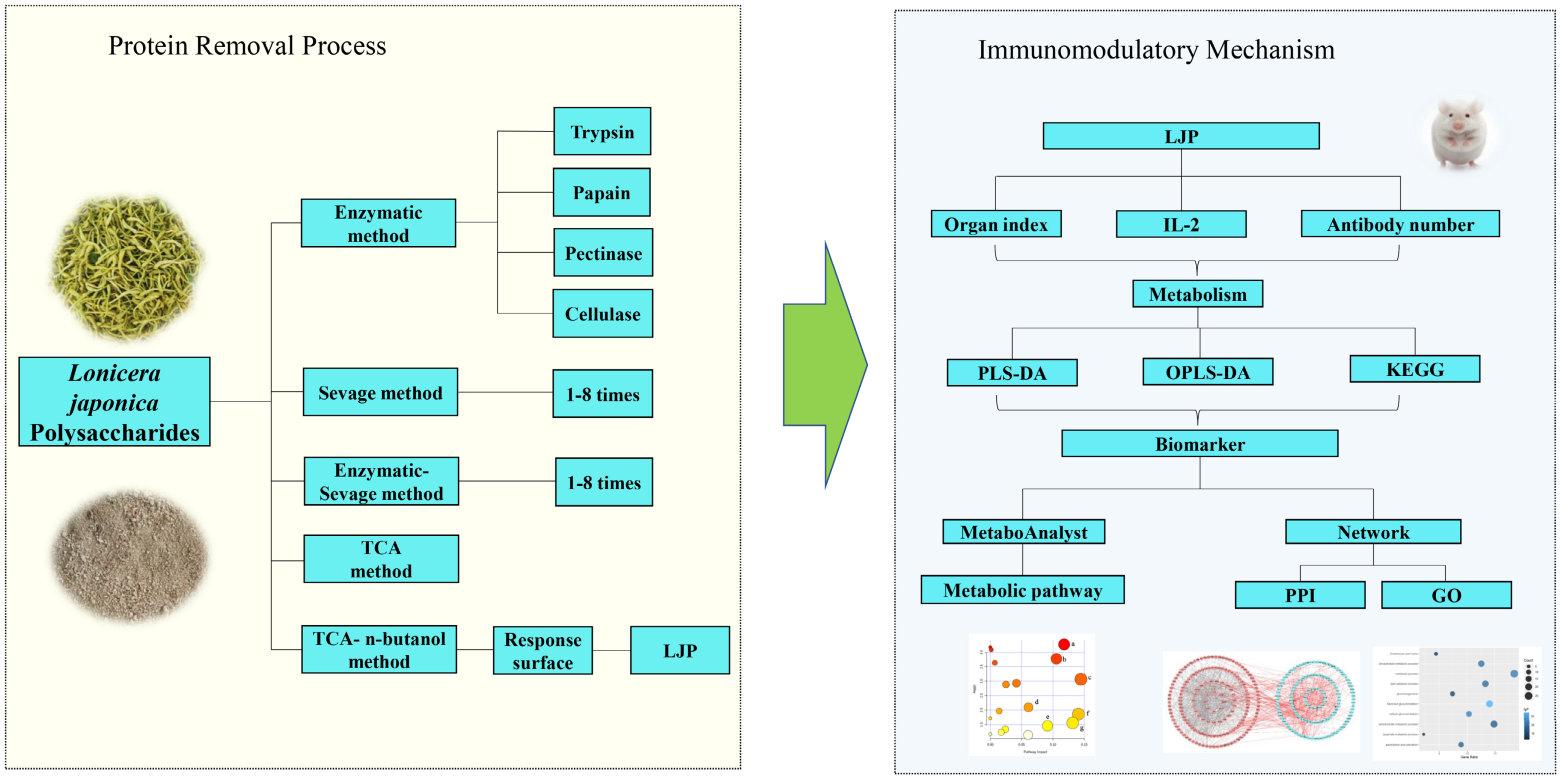
The process of the experiment

In this study, Kunming mice were used to establish a CTX‐induced immunodeficiency model and observe the immune enhancement effect of *Lonicera japonica* polysaccharides, and screen differential metabolites through serum metabolomics. In addition, we also combined network pharmacology to further explore the action mechanism of *Lonicera japonica* polysaccharides in immunomodulation. The process of the experiment is shown in Figure 1.

## 2 MATERIALS AND METHODS

### 2.1 Reagents and materials

*Lonicera japonica* Thunb. was purchased from BWT Chinese Herbal Medicine Slice Co., Ltd, and authenticated by Professor Jia Li in the School of Pharmaceutical Sciences, Shandong University of Traditional Chinese Medicine. Acetonitrile and methanol (chromatography grade) were purchased from Thermo Fisher Scientific Inc.

### 2.2 Extraction of *Lonicera japonica* crude polysaccharides

*Lonicera japonica* (1.0 kg) was extracted with distilled water (1:15, w/v) at 85°C for 2 h for 2 times. The supernatant was collected, concentrated, and added 4 volumes of absolute ethanol, then allowed to stand at 4°C overnight. The precipitate was collected and washed with absolute ethanol and diethyl ether, then dried under vacuum at 60°C. After that, the *Lonicera japonica* crude polysaccharide was obtained (Liu, Huang, & Hu, 2018; Liu, Liu, et al., 2015).

### 2.3 Comparison of *Lonicera japonica* crude polysaccharide removal methods

When we investigated the effect of removing protein, the phenol–sulfuric acid method and the Folin–phenol method were used to determine the content of polysaccharides and protein before and after protein removal (Cao et al., 2022). The polysaccharide retention rate, protein clearance rate, and comprehensive score were used as indicators to evaluate the effect of protein removal. The calculations were as follows:

$$\text{The polysaccharide retention rate}\;\left(\%\right)=m_{1}/m_{2}\times 100$$

where m_1_ is the polysaccharide weight of the *Lonicera japonica* crude polysaccharide after protein removal and m_2_ is the polysaccharide weight of the *Lonicera japonica* crude polysaccharide before protein removal.

$$\text{The protein clearance rate}\;\left(\%\right)=\left(1-m_{3}/m_{4}\right)\times 100$$

where m_3_ is the protein weight of the *Lonicera japonica* crude polysaccharide after protein removal and m_4_ is the protein weight of the *Lonicera japonica* crude polysaccharide before protein removal.

$$\text{The comprehensive score}=1/2\times \left(W_{1}/W_{1,\max}+W_{2}/W_{2,\max}\right)\times 100$$

where W_1_ is the polysaccharide retention rate, W_1,max_ is the maximum polysaccharide retention rate, W_2_ is the protein clearance rate, and W_2,max_ is the maximum protein clearance rate.

#### 2.3.1 Enzymatic protein removal method

Four equal parts of crude polysaccharide samples were taken and dissolved in distilled water, then added 0.4% trypsin, 0.9% papain, 1% pectinase, and 4.0% cellulase by mass of polysaccharide to remove protein. After reacting for 2 h at their optimal enzyme activity temperature and pH range, respectively, the samples were adjusted to pH 7 with 6 mol/L NaOH. The enzyme was inactivated by increasing the temperature. After that, the polysaccharide samples were centrifuged, ethanol precipitated, and collected. The content of polysaccharides and protein was determined to calculate the polysaccharide retention rate and protein clearance rate.

#### 2.3.2 Sevage protein removal method

Eight equal parts of crude polysaccharide samples were taken, dissolved in distilled water, and named 1 ~ 8. Added the 1/5 volume times Sevage reagent (trichloromethane:n‐butanol = 5: 1), vigorously shook for 20 min, then centrifuged (3500 r/min, 6 min, 4°C), and collected the upper layer. The other parts repeated this procedure 2 ~ 8 times. Then, the *Lonicera japonica* polysaccharides were precipitated with 4 volume absolute ethanol. After that, the polysaccharide retention rate and protein clearance rate were calculated.

#### 2.3.3 Enzymatic–Sevage protein removal method

The polysaccharide after removing protein by trypsin enzymolysis was taken, divided into eight parts, dissolved in distilled water, and named 1 ~ 8. The latter processes were similar to Sevage method and deproteinized eight parts for 1 ~ 8 times, respectively. Then the polysaccharide retention rate and protein clearance rate were calculated.

#### 2.3.4 TCA protein removal method

The *Lonicera japonica* crude polysaccharides were dissolved in distilled water, added the equal volume of 20% tricarboxylic acid (TCA) reagent, and intensely shook for 30 min. After waiting for 1 h at 4°C, the polysaccharide solution was centrifuged (3500 r/min, 6 min, 4°C), and adjusted to pH 7 with 6 mol/L NaOH, then precipitated and collected. The polysaccharide retention rate and protein clearance rate were calculated (Chen & Huang, 2018).

#### 2.3.5 TCA–n‐butanol protein removal method

Taken two equal *Lonicera japonica* crude polysaccharides and dissolved in distilled water, named 1 and 2. Added twice volume (20% TCA:n‐butanol = 1:10) reagent, shook for 15 min, and waited for 1 h at 4°C, collected the bottom layer and the two groups repeated this procedure 2 times. The polysaccharide was concentrated, precipitated, and dried and then calculated the polysaccharide retention rate and protein clearance rate.

### 2.4 Experimental design of RSM

A three‐factor, three‐level Box–Behnken design (BBD) designed with Design Expert Software was applied to optimize the best condition of deproteinization. Three independent variables, TCA: n‐butanol (v/v) (A), polysaccharide solution: (TCA–n‐butanol) (v/v) (B), and shaking time (min) (C), were used in the experimental design. The comprehensive score (Y) was regarded as the response. And the BBD is described in Table 1.

**TABLE 1:** Variables and levels in Box–Behnken design (BBD)

Variables	Coded	Coded levels		
		−1	0	1
TCA:n‐butanol (v/v)	A	1:5	1:10	1:15
Polysaccharide solution: (TCA–n‐butanol) (v/v)	B	1:1	1:2	1:3
Shaking time (min)	C	20	30	40

### 2.5 Animal

Twenty‐four male Kunming mice, weighing 22–28 g, were provided by Shandong Lukang Pharmaceutical Co., Ltd. Laboratory Animal Center (SCXK, 20140002, Jinan, China). All animal experiments were performed according to *the Guide for the Care and Use of Laboratory Animals* and approved by the Animal Ethics Committee of the Shandong University of Traditional Chinese Medicine. Sheep red blood cells (SRBC) were obtained from Nanjing constipation Biotechnology Co., Ltd, China. Mouse IL‐2 Enzyme‐Linked Immunosorbent Assay (ELISA) kit was provided by Assay Biotechnology (USA).

### 2.6 Experimental design

After two days of adaptive feeding, 24 mice were randomly divided into four groups (*n* = 6), including normal group, model group, LNT group (treated with 30 mg/kg lentinan, LNT), and LJP group (treated with 800 mg/kg polysaccharide which deproteinized protein with the best method and the best condition). All groups received intragastric administration of 10 ml/kg each time for three days, while the normal group and model group were given the same volume of physiological saline. Then all groups were intraperitoneally injected with 50 mg/kg cyclophosphamide (CTX) once a day for three consecutive days, except the control group treated with the same volume of physiological saline, to build the CTX immunocompromised mice. All mice were allowed free access to food and water during the experiment. After successful modeling, all groups were continued to be administered for 10 days.

### 2.7 Effect of LJP on mice organ index

After 12 h of the last treatment, the animals were weighed and blood was collected by removing the eyeballs, then the mice were sacrificed and collected the organs. The thymus and spleen were weighted and the indices were calculated as follows:

$$\text{Organ index}\;\left(\mathrm{mg}/g\right)=\text{the organ weight}\;\left(\mathrm{mg}\right)/\text{body weight}\;\left(g\right).$$

### 2.8 Effect of LJP on IL‐2 in serum and the level of serum hemolysin

On the seventh day of treatment, the mice began to be injected with 0.2 ml of 2% SRBC. After 4 days, the blood samples were collected from the eyes, then centrifuged, and the serum collected. Then the saline was used to multiple dilute the serum. Fifty microliters of 0.2% SRBC was added to the dilute mentioned above and incubated for 3 h at 37°C.

$$\text{The antibody number}=\left({\text{1L}}_{0}+{\text{2L}}_{1}+{\text{3L}}_{2}+\mathrm{XXX}+{\mathrm{nL}}_{n}\right)$$

where 1, 2, 3, …, n, are multiple dilute indices and Ln is the degree of agglutination.

The IL‐2 level in serum of mice was determined with ELISA kit according to the instructions provided by the manufacturer.

### 2.9 Metabolite sample collection

After 12 h of the last treatment, the eyeballs were removed and blood was collected, then the mice were sacrificed. The blood was left at room temperature for 0.5 h and then centrifuged (12,000 r/min, 15 min, 4°C) to isolate the serum and the plasma. The serum was collected, accurately transferred 150 μl, 450 μl methanol added, vortex‐mixed for 60 s, and centrifuged (12,000 r/min, 15 min, 4°C). Finally, the supernatant was injected into HPLC‐TOF‐MS (high‐performance liquid chromatography time‐of‐flight mass spectrometry) for analysis.

### 2.10 Chromatography conditions

Chromatographic analysis of serum samples was performed on the Halo‐C_18_ column (2.1 × 100 mm, 2.7 μm, Waters, USA). The column temperature was 35°C while the flow rate was 0.25 ml/min. The injection volume was 5 μl. The mobile phase used 0.1% aqueous formic acid (A) and acetonitrile (B). The gradient elution was as follows: 20%–85% B over 0–10 min, 85%–100% B over 11–20 min, and 100% B over 21–30 min. Mass spectrometry conditions were set as follows: the capillary voltage was 4000 V, the drying gas temperature was 350°C, and the gas flow rate was 8 L/min. The mass collection range was 50–1000 (Zhang et al., 2018).

### 2.11 Data processing and pathway analysis

Agilent Qualitative Analysis was used to extract the data, identify and integrate chromatographic peaks. The peaks aligned and matched were used by Mass Profiler. Multivariate data (MVD) analysis was performed by the supervised learning method with the partial least‐squares discriminant analysis (PLS‐DA) and orthogonal partial least‐squares discriminant analysis (OPLS‐DA) by using SIMCA‐P 13.0 (Umetrics, Umea, Sweden). Biomarkers were discovered by screening for metabolic differences (Wang et al., 2019). The value of variable importance (VIP) in the project was used to rank the overall contribution of each variable in OPLS‐DA (Jiang et al., 2019). VIP >1.0, FC >2, or FC <0.5, and *p* value < .05 were used to select the potential biomarkers. Meanwhile, Human Metabolome Database (HMDB) (https://hmdb.ca/) and Kyoto Encyclopedia of Genes and Genomes (KEGG) (https://www.kegg.jp/) databases were used to further identify the biomarkers. MetaboAnalyst 5.0 (https://www.metaboanalyst.ca/) was combined to construct the endogenous biomarkers' analytical metabolic pathway.

### 2.12 Network pharmacology analysis

In order to further analyze the affection of biomarkers, MetScape for Cytoscape software, which can integrate biomolecular interaction networks with high‐throughput expression data into a unified KEGG‐based conceptual framework, was used to dig the upstream proteins of biomarkers. Meanwhile, the proteins related to immunomodulatory were collected based on the GeneCards database (https://www.genecards.org/). These upstream proteins and pathological proteins were imported into STRING for protein–protein interactions analysis (PPI). The minimum required interaction score was set at 0.9 and hid disconnected nodes in the network to link their interactions. In this network, every node stood for a protein, and the edges stood for their relationship. For analyzing the synthetic biological functional annotation information, the collected proteins were connected to their official gene symbol (UniProt ID) and imported into the Database for Annotation, Visualization and Integrated Discovery (DAVID) (https://david.ncifcrf.gov/), which could find the most significant enrichment biological annotation, selected the *Homo sapiens*. Based on the Gene Ontology (GO) analysis, the genes molecular functions information was determined. Data visualization was carried out with a threshold (*p* < .05).

### 2.13 Statistical analysis

All data obtained were summarized as mean ± standard deviation (SD) and statistical testing and comparisons were performed by using the one‐way analysis of variance (ANOVA) method. Statistical analyses were done using SPSS 21.0. A value of *p* < .05 was considered as statistical significance.

## 3 RESULTS

### 3.1 Deproteinization results’ analysis of five methods

**FIGURE 2:**
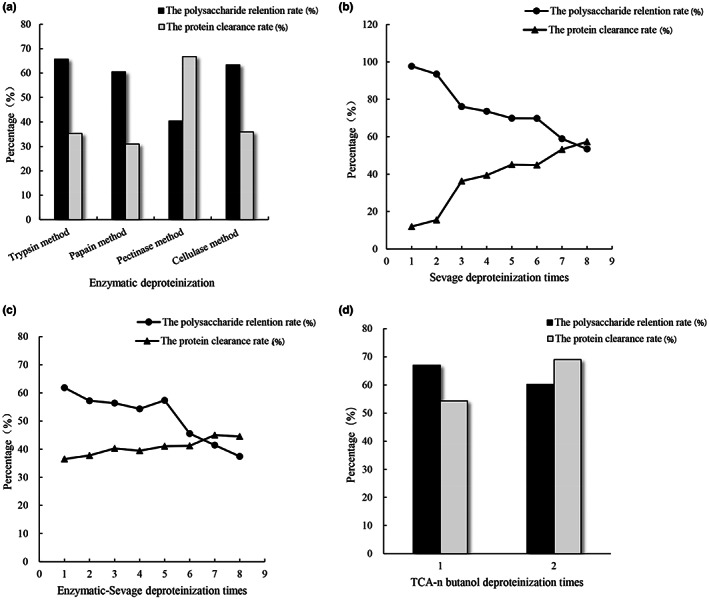
The polysaccharide retention rate and the protein clearance rate of polysaccharide deproteinized by the (a) enzymatic method, (b) Sevage method, (c) Enzymatic–Sevage method, and (d) TCA–n‐butanol method, respectively

*Lonicera japonica* polysaccharides were deproteinized by the enzymatic method, Sevage method, enzymatic–Sevage method, TCA method, and TCA–n‐butanol method, respectively. Considering the high polysaccharide retention rate and protein clearance rate, the trypsin method could be used for deproteinization as the best enzymatic method (Figure 2a). The results of the Sevage method are shown in Figure 2b, indicating that the appropriate deproteinization times were 6. The trypsin method combined with the Sevage method was also used to remove the protein (Figure 2c). To obtain high polysaccharide retention rate and protein clearance, 5 times of deproteinization was the appropriate time in the Trypsin–Sevage method. After using the TCA method, although the protein removal effect was ideal which reached 65.13%, the polysaccharide retention rate was only 39.32%, which may be due to more evident damage caused by TCA to *Lonicera japonica* polysaccharides. As presented in Figure 2d, after the protein was removed with TCA–n‐butanol two times, the protein clearance rate increased, but the polysaccharide retention rate decreased. Considering the simplicity of the operation process, the frequency of protein removal with TCA–n‐butanol was once.

### 3.2 Comparison of the five methods for deproteinization

**FIGURE 3:**
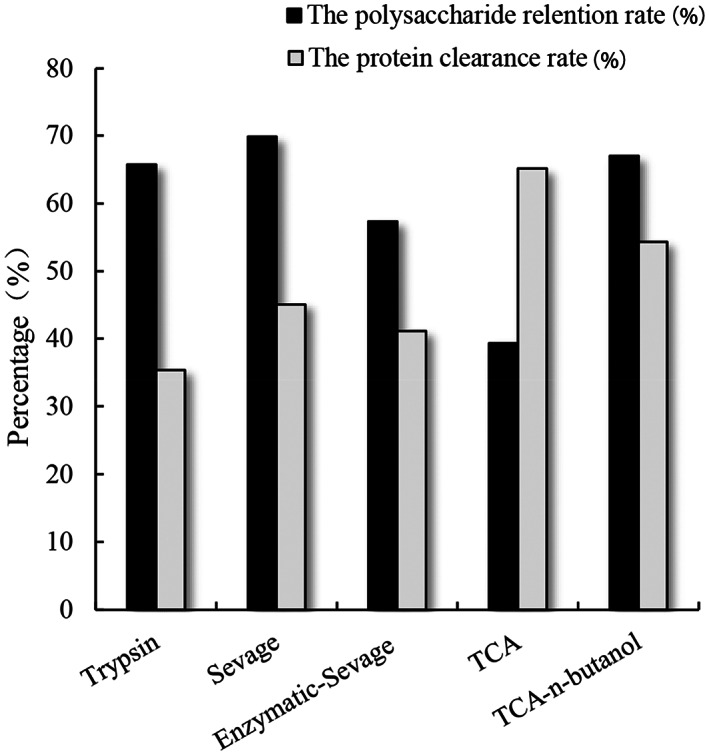
Comparison of five methods’ best results for deproteinization

As shown in Figure 3, the best protein removal results in each method of Enzymatic, Sevage, Enzymatic–Sevage, TCA, and TCA–n‐butanol methods were compared. The polysaccharide retention rates of them were 65.72%, 69.83%, 57.35%, 39.32%, and 67.00%, while their protein clearance rates were 35.37%, 45.09%, 41.09%, 65.13%, and 54.34%, respectively. Among the above‐mentioned five deproteinization methods, TCA–n‐butanol method exhibited a high protein clearance rate and polysaccharide retention rate, indicating that TCA–n‐butanol method was the suitable protein removal method for *Lonicera japonica* polysaccharides.

### 3.3 Optimization of the deproteination by RSM

#### 3.3.1 Statistical analysis and model fitting

Response surface methodology (RSM) is a collection of statistical and mathematical techniques useful for developing, improving, and optimizing processes (Liu, Ma, et al., 2015). A total of 17 designed experiments with three factors and three levels were conducted for optimizing the deproteinization conditions (Table 2). The experiment data were analyzed by multiple regression analysis, and the second‐order polynomial equation was as follows:

$$\begin{array}{c} Y=86.77-1.71A+3.47B+0.75C-1.49\mathrm{AB}+1.07\mathrm{AC}-2.57\mathrm{BC}\\ \;+1.32A^{2}-3.48B^{2}-1.12C^{2} \end{array}$$

**TABLE 2:** Response surface experiment design and results

No.	A (TCA: n‐butanol, v/v)	B (Polysaccharide solution: (TCA–n‐butanol), v/v)	C (Shaking time, min)	Y (The comprehensive score)
1	0	0	0	86.60
2	0	0	0	84.83
3	−1	1	0	90.37
4	−1	0	−1	90.29
5	0	1	1	84.16
6	1	−1	0	81.85
7	1	0	−1	82.87
8	−1	−1	0	80.41
9	0	0	0	86.77
10	−1	0	1	88.95
11	0	−1	−1	75.04
12	0	1	−1	87.10
13	0	−1	1	82.40
14	0	0	0	88.41
15	0	0	0	87.25
16	1	0	1	85.79
17	1	1	0	85.85

The analysis of variance (ANOVA) of the regression model is shown in Table 3. The importance of the coefficient model was measured by the F‐value and *p*‐value (Guo et al., 2019). The model F‐value was 11.97, and the corresponding *p*‐value was .0018 < .01, demonstrating that the model was fitted well. The *F*‐value of lack of fit was 1.60 and *p*‐value was .32 > .05, which illustrated the validity of the model. The results showed that the factors A, B, B^2^, and the interaction item BC were significant (*p* < .05), indicating that the factors were not a simple linear correlation. The order of the influence of single factors on the comprehensive score was polysaccharide solution: (TCA–n‐butanol) (B) > TCA: n‐butanol (A) > shaking time (C).

**TABLE 3:** Regression model analysis of variance (ANOVA)

Source	Sum of squares	Df	Mean square	*F* value	*p*‐value Prob > *F*	Significant
Model	227.12	9	25.24	11.97	.0018	**
A	23.32	1	23.32	11.07	.0127	*
B	96.47	1	96.47	45.77	.0003	**
C	4.50	1	4.50	2.13	.1874	
AB	8.88	1	8.88	4.21	.0792	
AC	4.54	1	4.54	2.15	.1858	
BC	26.52	1	26.52	12.58	.0094	**
A^2^	7.38	1	7.38	3.50	.1035	
B^2^	50.87	1	50.87	24.14	.0017	**
C^2^	5.29	1	5.29	2.51	.1571	
Residual	14.75	7	2.11			
Lack of Fit	8.04	3	2.68	1.60	.3230	
Pure Error	6.71	4	1.68			
Cor Total	241.87	16				

*Note*: *significant difference (*p* < .05), **extreme significant difference (*p* < .01).

#### 3.3.2 Response surface analysis

**FIGURE 4:**
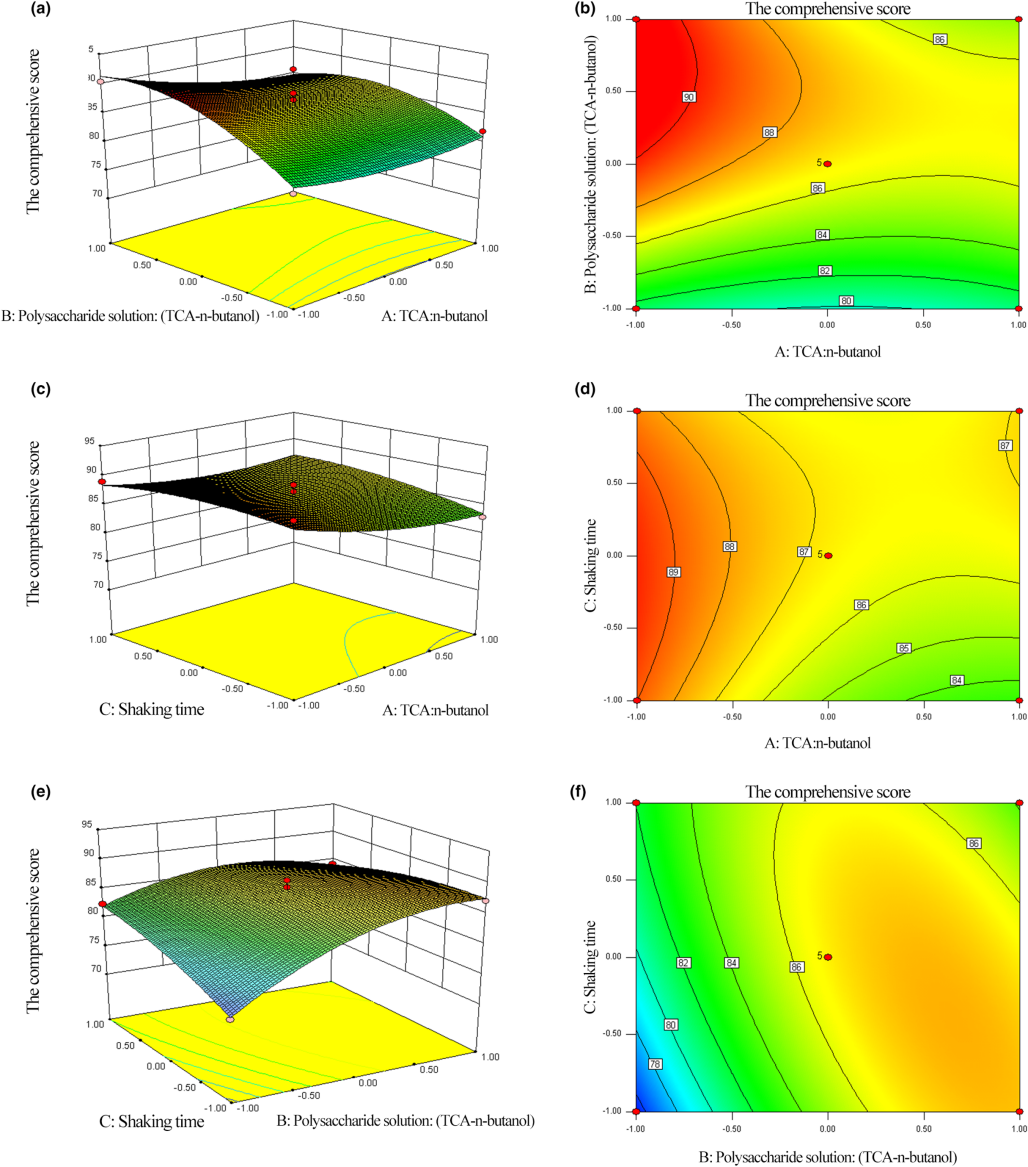
Three‐dimensional (3D) response surface plots (a, c, e) and two‐dimensional (2D) contour plots (b, d, f) of three variables on *Lonicera japonica* polysaccharides’ deproteinization. (a, b) The interaction between TCA:n‐butanol and polysaccharide solution:(TCA–n‐butanol); (c, d) The interaction between TCA:n‐butanol and shaking time; (e, f) The interaction between polysaccharide solution: (TCA–n‐butanol) and shaking time

To further explain the interaction between the independent variables and response values, three‐dimensional (3D) response surface plots and two‐dimensional (2D) contour plots are displayed in Figure 4. By analyzing these 3D response surface plots and their respective profiles, it was easy to understand the interaction between two independent variables and determine their optimal range (Liu, Ma, et al., 2015). As demonstrated in Figure 4, the polysaccharide solution: (TCA–n‐butanol) had a significant effect on the comprehensive score. Meanwhile, the results showed that the polysaccharide solution: (TCA–n‐butanol) and the shaking time have complex interaction effects.

#### 3.3.3 Model verification

By analyzing these data, the maximum comprehensive score of 90.75 was obtained at the following optimum conditions: TCA:n‐butanol = 1:5.05, polysaccharide solution: (TCA–n‐butanol) = 1: 2.78, and shook for 32.90 min. For the convenience of operation, the TCA:n‐butanol was 1:5.1, the polysaccharide solution: (TCA–n‐butanol) was 1:2.8, and shook for 33 min. To verify this condition, three confirmation experiments were performed, and the average comprehensive score was 91.80, which was close to the predicted value. This result proved the validity of the prediction. Under the optimal condition, LJP was prepared and its immunomodulatory activity was further investigated.

### 3.4 Immunological effect of LJP on CTX‐induced mice

#### 3.4.1 Immune organ index

**FIGURE 5:**
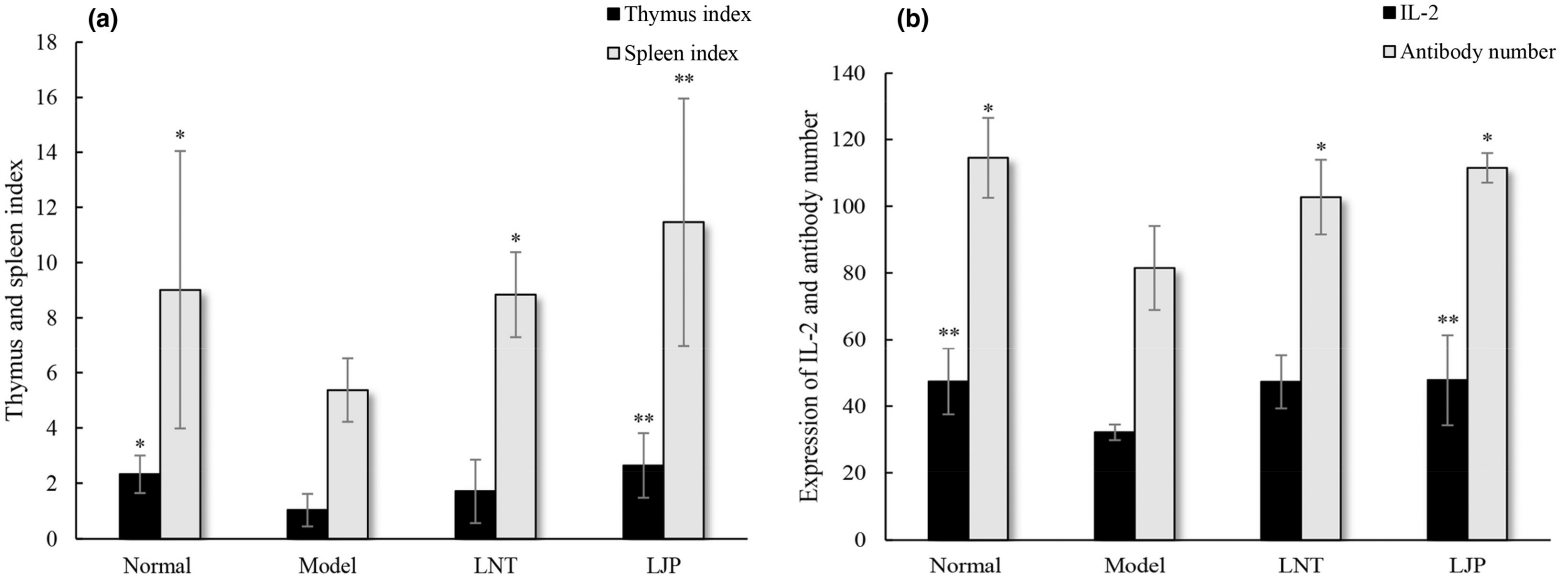
(a) Effect of *Lonicera japonica* polysaccharide (LJP) on immune organ index in mice. The values are presented as mean ± S.D. *p < .05, **p < .01, compared with model group. (b) The effect of LJP on interleukin 2 (IL‐2) level and the antibody number. The values are presented as mean ± S.D. *p < .05, **p < .01, compared with the model group

The thymus and spleen are the main immune organs of the body, which affect the organism's immune function and the ability to resist disease. According to Figure 5a, the spleen and thymus indexes in the model group were much lower than those in the control group which illustrated that a weakened immune system was induced by CTX. After treatment with LJP, the spleen and thymus indexes were observably ameliorated, suggesting that LJP had a good immune‐enhancing effect on the recovery of CTX damage to immune organs.

#### 3.4.2 IL‐2 and serum hemolysin level

The immune response associated with levels of cytokines is a multistep process. IL‐2 is an important cytokine that directly affects cellular immunity and humoral immunity. Under certain conditions, serum hemolysin would combine with SRBC and cause SRBC hemolysis (Dai et al., 2021). To further investigate the effect of LJP on humoral immunity, serum hemolysin level was also measured, and the results were presented as the antibody number. As shown in Figure 5b, the serum levels of IL‐2 and antibody numbers in LJP group were significantly augmented compared with model group, suggesting that LJP could increase the secretion of IL‐2 cytokine and reduce the severity of immunosuppression in mice.

### 3.5 Metabolomic results

#### 3.5.1 Multivariate data analysis

**FIGURE 6:**
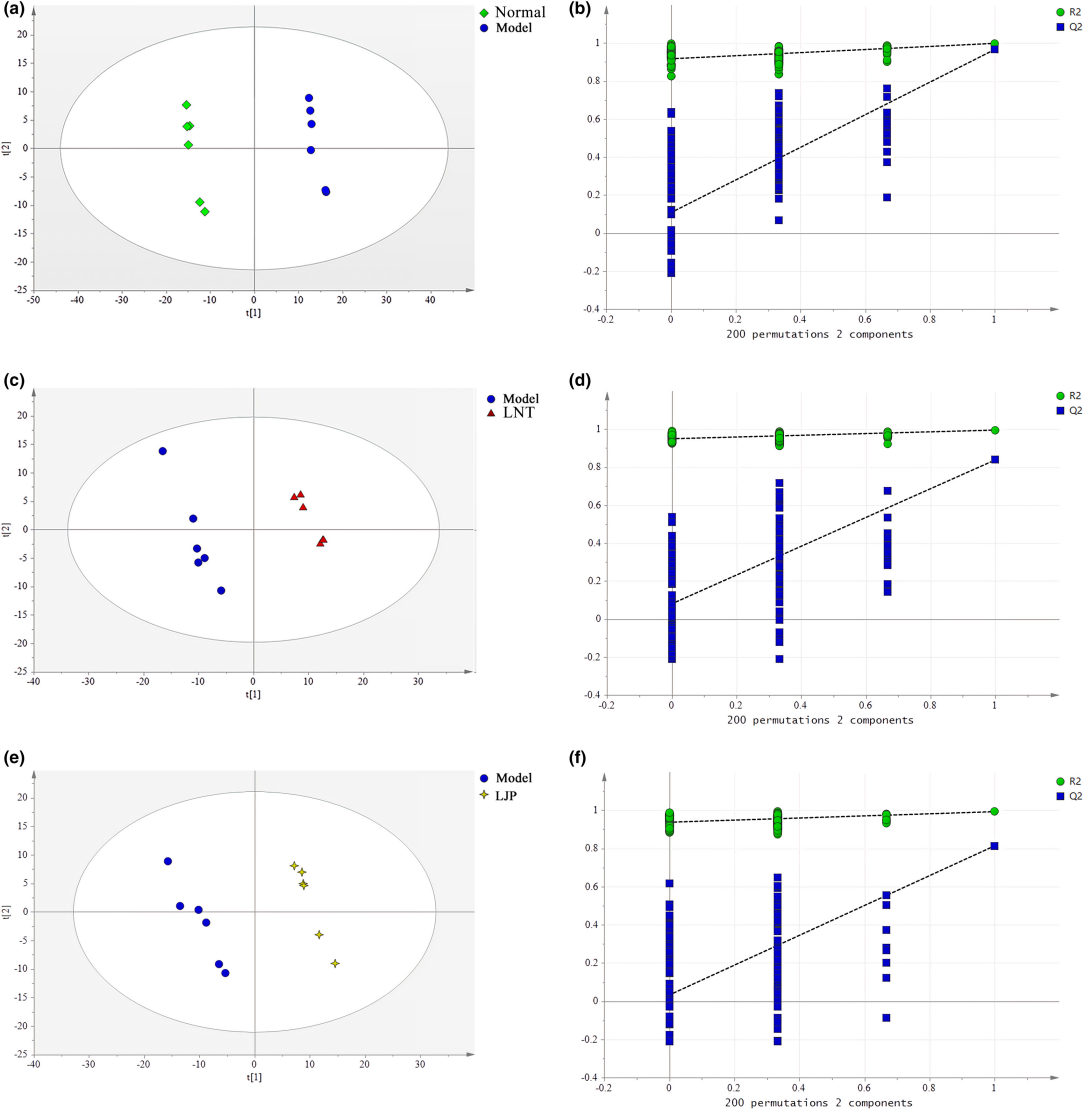
The results of partial least‐squares discriminant analysis (PLS‐DA) score plots and 200‐permutation tests. (a, b) PLS‐DA score plot and 200‐permutation tests for normal and model group (R^2^X = 0.470, R^2^Y = 0.999, Q^2^ = 0.966); (c, d) PLS‐DA score plot and 200‐permutation tests for model and LNT group (R^2^X = 0.308, R^2^Y = 0.995, Q^2^ = 0.838); (e, f) PLS‐DA score plot and 200‐permutation tests for model and LJP group (R^2^X = 0.310, R^2^Y = 0.993, Q^2^ = 0.814)

In this study, HPLC‐TOF‐MS method combined with SIMCA 13.0 was applied to analyze the serum of mice from normal, model, LNT, and LJP group. To further discover the differences among endogenous metabolites in each group, multivariate statistical analyses, including PLS‐DA and OPLS‐DA, were used (Hao et al., 2018). As shown in PLS‐DA score plots’ results (Figure 6a,c,e), the samples from the same group clustered together, and different groups' samples were better distinguished, showing good predictive ability. They also confirmed that the normal, model, LNT, and LJP group underwent metabolic changes. The permutation tests (*n* = 200) were used to validate the predictive ability of the PLS‐DA model (Figure 6b,d,f). The results showed that the experimental values of R^2^ and Q^2^ on the left side were always lower than the original values on the right, indicating that the results were not overfitting and that PLS‐DA models had better predictive capability.

**FIGURE 7:**
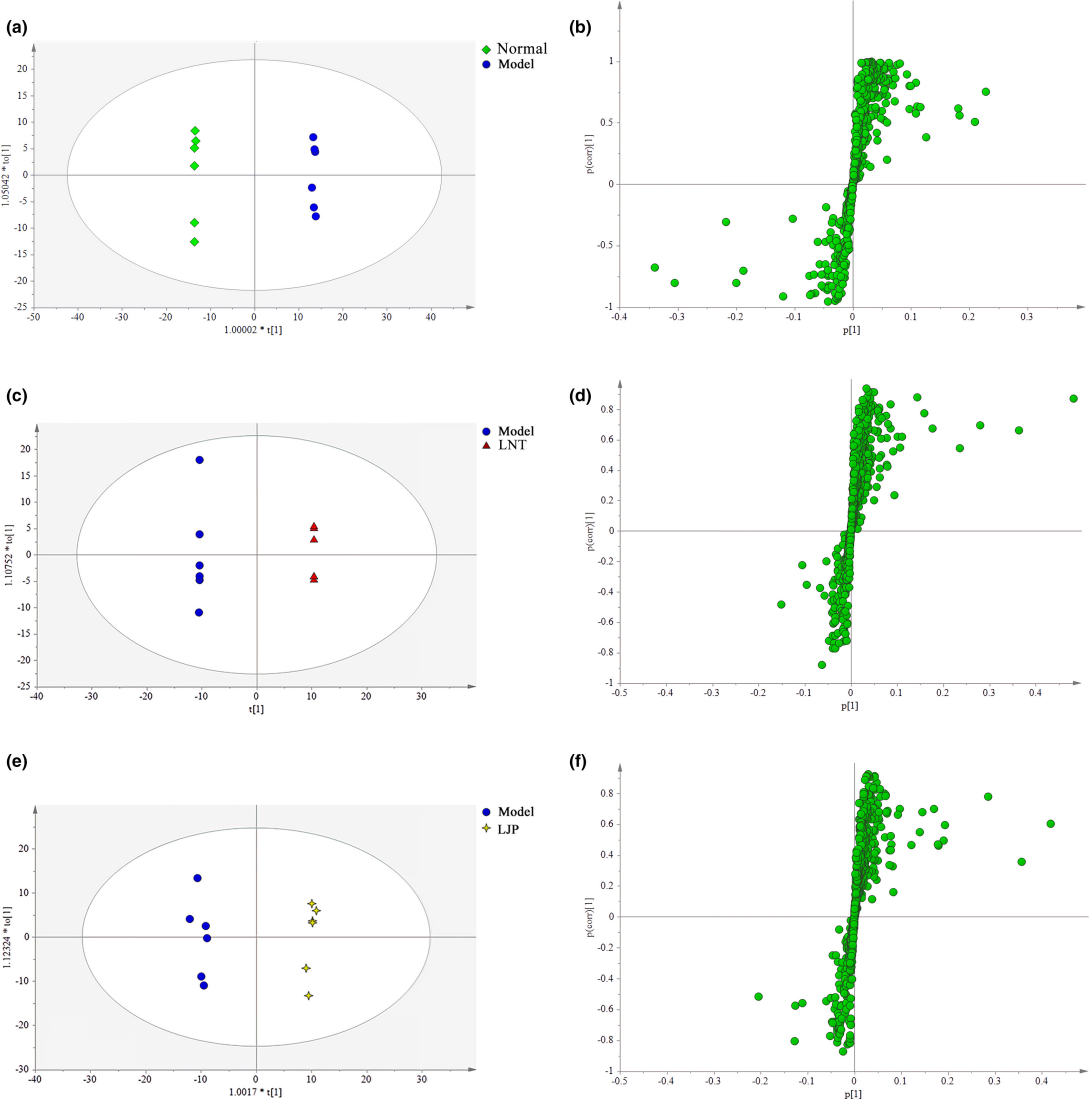
The results of orthogonal partial least‐squares discriminant analysis (OPLS‐DA) score plots and S‐plot. (a, b) OPLS‐DA score plots and S‐plot for normal and model group (R^2^X = 0.375, R^2^Y = 0987, Q^2^ = 0.945); (c, d) OPLS‐DA score plots and S‐plot for model and lentinan (LNT) group (R^2^X = 0.659, R^2^Y = 1, Q^2^ = 0.879); (e, f) OPLS‐DA score plots and S‐plot for model and *Lonicera japonica* polysaccharide (LJP) group (R^2^X = 0.310, R^2^Y = 0.993, Q^2^ = 0.775)

The OPLS‐DA was based on the verification of the PLS‐DA model. Without reducing the predictive ability of the model, OPLS‐DA could reduce the complexity of the model and enhance the explanatory ability of the model. According to the results of OPLS‐DA score plots, those points were separated into two groups, showing that this method could be remarkably distinguished between the metabolite profiles of the two groups (Figure 7a,c,e). In the S‐plot (Figure 7b,d,f), every point represented an ion part. The farther data point from the center made greater contributions to the separation of the two different groups; thus, those points were identified as specific metabolites.

#### 3.5.2 Metabolites’ identification

Potential biomarkers were selected by VIP, fold change (FC), and *p*‐value. The VIP >1.0 indicated that the variables' contribution to the model was above average (Yu et al., 2020). Meanwhile, only *p* < .05 was considered significant, so the 14 potential biomarkers with VIP >1.0, FC >2 or FC <0.5, and *p* value < .05 were finally chosen (Table 4). The changes of these metabolites in serum reflect the alteration of metabolic phenotype. Comparing normal and model group, 9 endogenous metabolites were upregulated and 5 endogenous metabolites were downregulated. After LJP treatment, all metabolites were recalled to some extent, indicating that LJP had a therapeutic effect on immunosuppressive mice.

**TABLE 4:** Statistical analysis results of potential biomarkers in serum

ID	Query mass	Biomarkers	KEGG	Formula	VIP	Change Tend	
						Model/Normal	LJP/ model
1	96.9991	Gentisic acid	C00628	C_7_H_6_O_4_	1.17183	↑	↓
2	317.9013	TG (16:0/16:0/18:0)	C00422	C_57_H_104_O_6_	1.27291	↓	↑
3	78.9831	Phosphoenolpyruvic acid	C00074	C_3_H_5_O_6_P	1.28033	↓	↑
4	211.0123	Oxalacetic acid	C00036	C_4_H_4_O_5_	1.34694	↓	↑
5	125.0946	Tryptamine	C00398	C_10_H_12_N_2_	1.43134	↑	↓
6	155.0691	Ornithine	C00077	C_5_H_12_N_2_O_2_	1.42361	↑	↓
7	224.1877	Spermidine	C00315	C_7_H_19_N_3_	1.32283	↓	↑
8	549.2986	LysoPC (18:1[9Z])	C04230	C_22_H_44_NO_7_P	1.40552	↓	↑
9	286.6264	SM (d18:1/18:0)	C00550	C_47_H_93_N_2_O_6_P	1.60415	↑	↓
10	929.4557	3‐Methoxy‐4‐hydroxyphenylglycol glucuronide	C03033	C_25_H_36_O_8_	1.36964	↑	↓
11	929.1592	2‐Methylacetoacetyl‐CoA	C03344	C_26_H_42_N_7_O_18_P_3_S	1.31803	↑	↓
12	929.5551	3, 5‐Diiodo‐L‐tyrosine	C01060	C_9_H_9_I_2_NO_3_	1.34041	↑	↓
13	462.1227	Glutaryl‐CoA	C00527	C_26_H_42_N_7_O_19_P_3_S	1.30257	↑	↓
14	330.2763	Lactosylceramide (d18:1/12:0)	C01290	C_55_H_105_NO_13_	1.46406	↑	↓

*Note*: The levels of differential metabolites are marked with downregulated (↓) and upregulated (↑).

#### 3.5.3 Metabolic pathway

**FIGURE 8:**
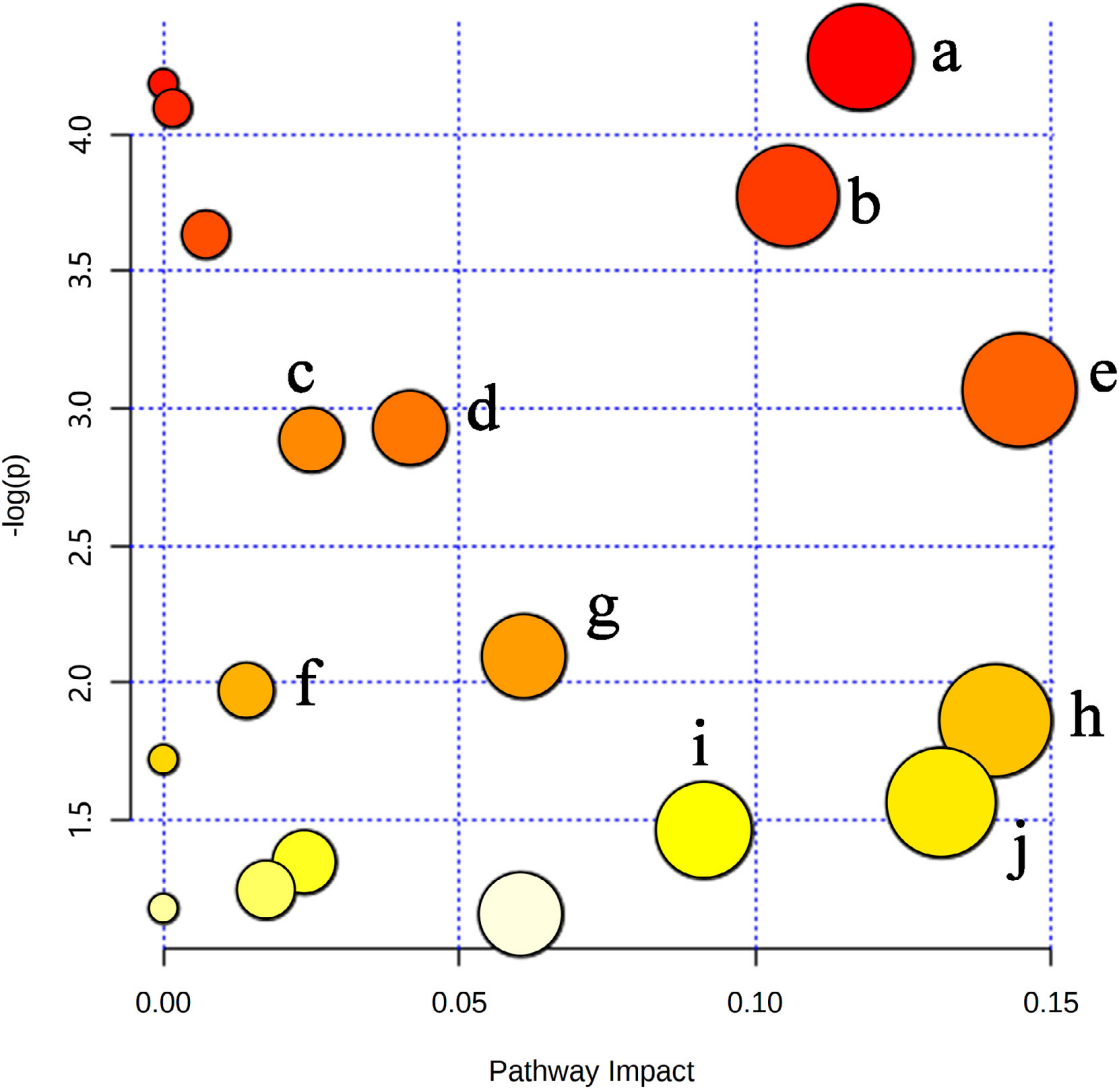
Summary of Pathway Analysis. (a) Citrate cycle; (b) Glycolysis / Gluconeogenesis; (c) Arginine and proline metabolism; (d) Arginine biosynthesis; (e) Alanine, aspartate, and glutamate metabolism; (f) Pentose and glucuronate interconversions; and (g) Lysine degradation

To further explore the metabolic pathways of potential biomarkers related to LJP in the treatment of immunocompromised mice, MetaboAnalyst 5.0 was used to perform the pathway enrichment analysis against the potential biomarkers. As shown in Figure 8, the x‐axis (pathway impact) stood for the importance of the metabolic pathway, and the y‐axis (−log P) stood for the significance of the metabolic pathway enrichment analysis. The KEGG numbers of the 14 biomarkers were imported into MetaboAnalyst 5.0, and the impact value threshold of pathway topology analysis was set to 0.06, the values above this threshold were chosen as potential target pathways. Seven main influential metabolic pathways were identified: Citrate cycle; Glycolysis/Gluconeogenesis; Arginine and proline metabolism; Arginine biosynthesis; Alanine, aspartate and glutamate metabolism; Pentose and glucuronate interconversions; Lysine degradation.

### 3.6 Network pharmacology results

**FIGURE 9:**
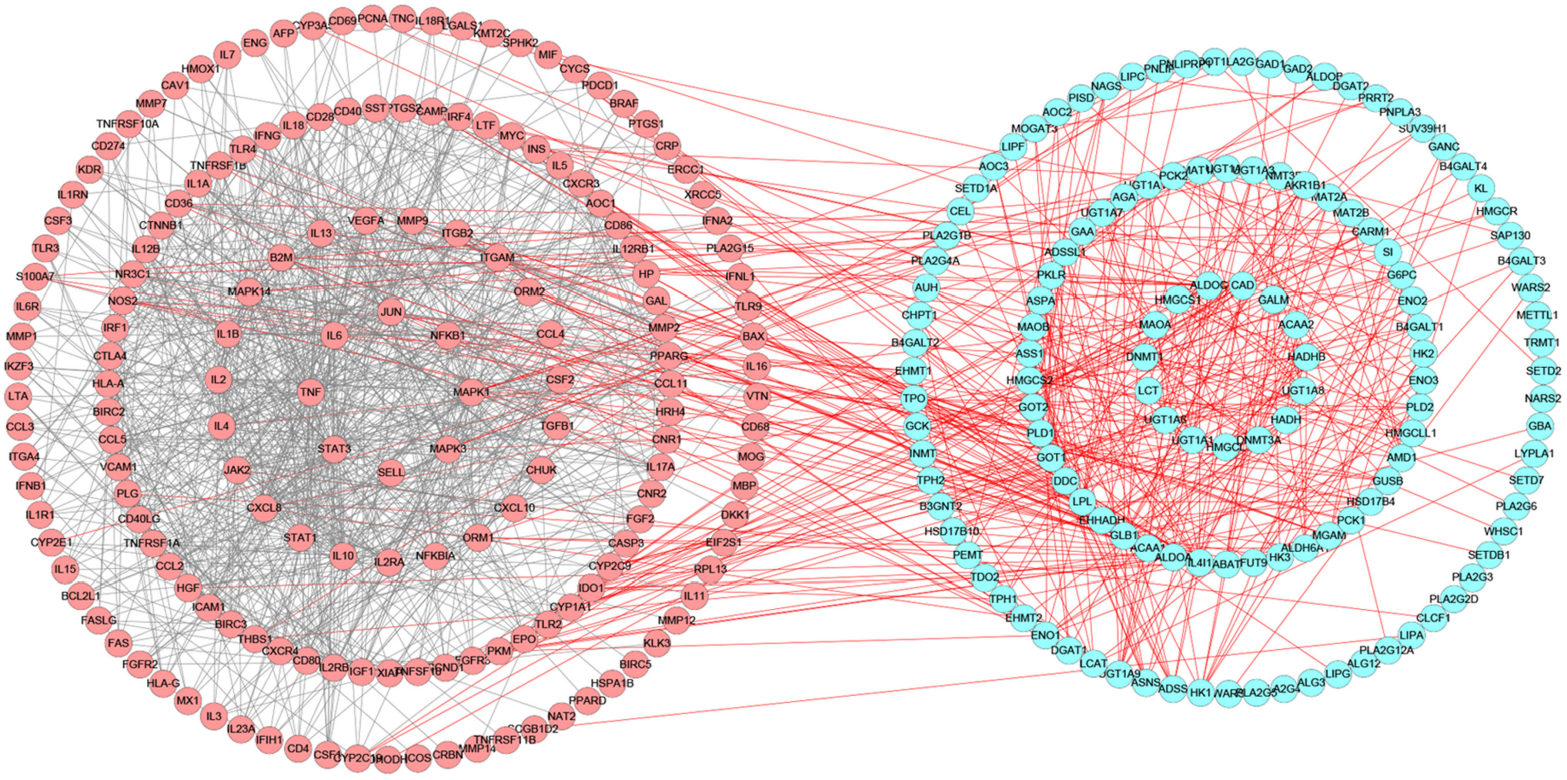
Protein–protein interactions analysis (PPI) network. The red and blue nodes represent the immune‐related targets and potential biomarker‐related targets, respectively. The red lines stand for the interaction between immune‐related targets and potential biomarkers, while the black lines indicate the interaction between immune‐related targets

To further illustrate the potential mechanism of LJP on immunomodulatory effect, PPI was used to analyze the correlation between metabolic pathways and corresponding targets. As displayed in Figure 9, 132 upstream proteins were collected by MetScape based on the related metabolic targets, and 171 immunodeficiency targeting proteins were selected through GeneCard database. Meanwhile, there were 303 nodes and 1306 edges in the network, and the more edges showed the stronger the interaction. According to the network, the connection between upstream targets and disease targets could be found, which indirectly proved the metabolomics results (Liu et al., 2019). Among the 132 upstream proteins, 57 key targeting proteins were selected to analyze the biological functions during the immunodeficiency progress, which could link more target proteins related to immunodeficiency.

**FIGURE 10:**
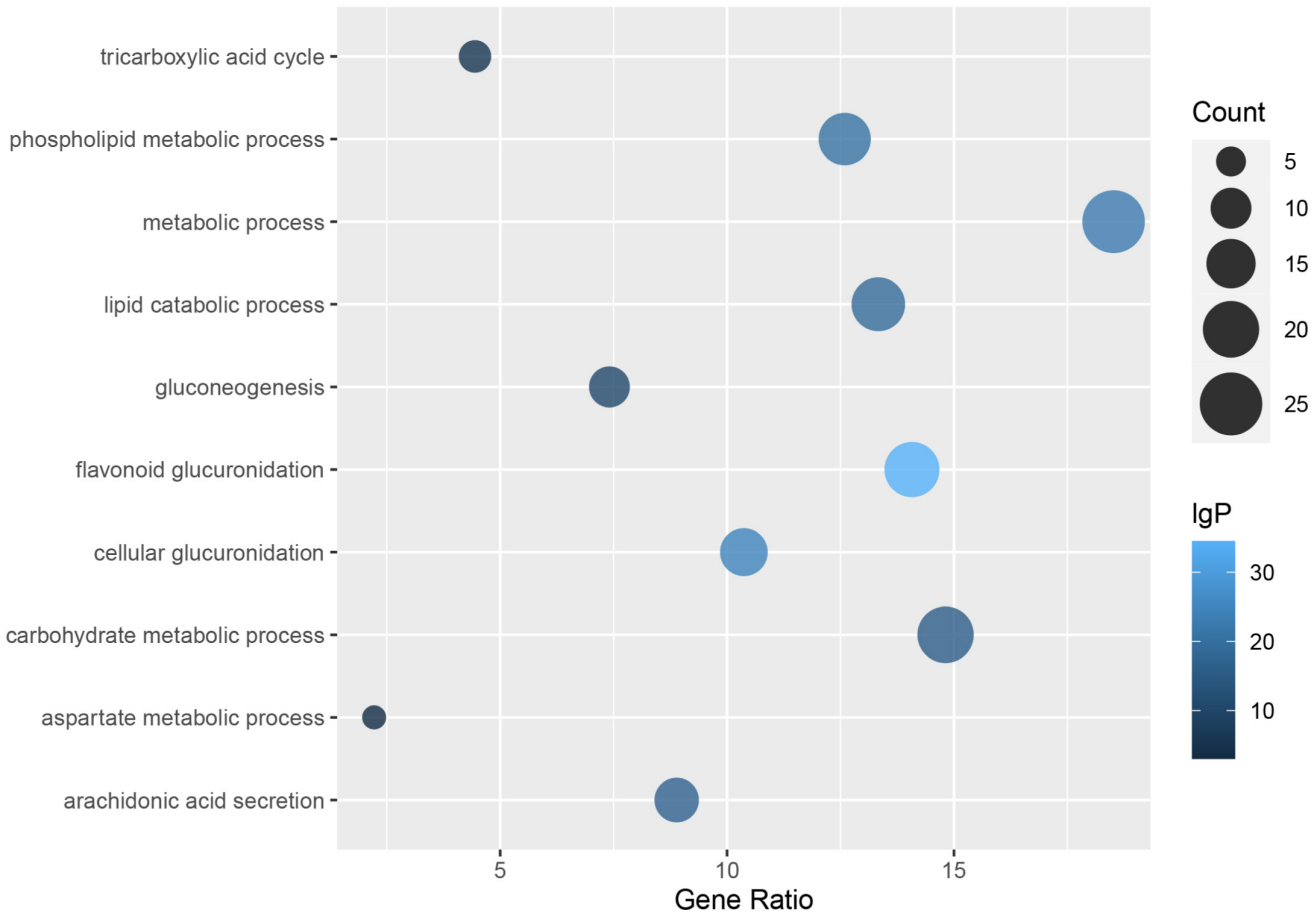
The biological functions associated with the immunomodulatory‐related targeted proteins and the potential biomarkers

Figure 10 explains the relationship between important biological functions and their gene ratio. The results showed that the biological functions relate to the immunomodulatory‐related targeted proteins and the potential biomarkers, including tricarboxylic acid cycle, phospholipid metabolic process, metabolic process, lipid catabolic process, gluconeogenesis, flavonoid glucuronidation, cellular glucuronidation, carbohydrate metabolic process, aspartate metabolic process, and arachidonic acid secretion.

## 4 DISCUSSION

As one of the oldest medicine systems in the world, TCM has the characteristics of multiple components and multiple targets and has shown great advantages in the treatment of various diseases (Yang et al., 2021). Some reports showed that as the important active ingredient of TCM, herbal polysaccharides have the characteristics of low toxicity and low side effects, and participate in many biological functions, such as antiviral, antitumor, antioxidant, hypolipidemic, immune regulation, and so on. Therefore, the separation, purification, and biological activity of TCM polysaccharides have become a research hotspot at home and abroad (Zeng et al., 2019).

*Lonicera japonica* polysaccharides were used in the long course of medical practice. However, the polysaccharides extracted by traditional methods were usually impure and contained much protein, which affected the judgment of the activity of polysaccharides.

In this work, we investigated five methods to remove proteins: Enzymatic method, Sevage method, Enzymatic–Sevage, TCA method, and TCA–n‐butanol method. We comprehensively explored the protein removal methods of *Lonicera japonica* polysaccharides from two aspects: the selection of deproteinized reagents and the number of deproteinized times. By comparing the best results of each method, it was determined that TCA–n‐butanol method was the best method. In order to obtain better results of protein removal, we optimized the process by RSM. Based on the experimental data, the best protein removal condition was TCA:n‐butanol = 1: 5.1, polysaccharide solution: (TCA–n‐butanol) = 1:2.8, and shook for 33 min.

Thymus and spleen are the main central immune organs and the largest peripheral immune organs, respectively (Dai et al., 2021). Both of them can directly reflect the level of immune function (Lv et al., 2019). The results showed that LJP could significantly promote the thymus and spleen indexes of mice. Interleukin‐2 (IL‐2) is a kind of T‐cell growth factor activity found in the supernatant of activated T cells in 1976 and is the main cytokine in the stimulation of T lymphocyte proliferation (Chen et al., 2006; Liao et al., 2011). Serum hemolysin level is the main indicator reflecting nonspecific immune function, which reflects the proliferation and differentiation of B cells and their ability to secrete hemolysin after combining with complement (Wang et al., 2020). To investigate the effect of LJP on the immunosuppressed complement system, the level of IL‐2 and serum hemolysin was measured. The results showed that the content of IL‐2 and serum hemolysin was noticeably ameliorated after the LJP treatment.

Metabolomic is an emerging method that can reveal the specific changes in phenotypic physiological and biochemical states in biological systems. Hence, immunometabolism, notably amino acids, fatty acids, and glucose, is essentially needed for immune cells in homeostasis or pathological state as a new branch of metabolism. The metabolites of some acids, such as tryptophan, are related to cell proliferation and growth processes. Amino acids play a crucial role as regulators and as main substrates in a variety of metabolic pathways (Babu et al., 2019). The upregulation of Glycolysis was a critical step in the activation of innate and adaptive immune cells, as it provides a means of increasing flux through the pentose phosphate pathway to synthesize macromolecules and generate the antimicrobial respiratory burst (Ganeshan & Chawla, 2014). Some reports showed that increased glycolysis could be considered a sign of metabolic changes in the rapid activation of most immune cells (O'Neill et al., 2016). Notably, existing reviews show that amino acid metabolism plays an important role in regulating the immune system. According to the metabolomics methods, pathways of differential metabolites in the LJP for the treatment of immunosuppressed mice were Citrate cycle, Glycolysis/Gluconeogenesis, and so on. Compared with the model group, LJP adjusted these main pathways to gradually restore the normal level of immunocompromised mice. At the same time, it was the first time to elucidate the mechanisms of action of LJP in immune regulation by the approach of network pharmacology. Nonetheless, further experiments are still needed to validate our findings.

## 5 CONCLUSION

In short, through optimization of conditions, we determined that the best method for removing protein of LJP was the TCA–n‐butanol method, and the best condition was TCA:n‐butanol = 1:5.1, polysaccharide solution: (TCA–n‐butanol) = 1:2.8, and shook for 33 min. After the CTX‐induced mice were treated with LJP, the thymus and spleen indexes were promoted, and the contents of IL‐2 and hemolysin in mice serum were also restored. Based on the serum metabolomics analysis, a total of 14 potential biomarkers about immune and LJP treatment have been identified in different groups. Taking these metabolites as the potential molecular targets, LJP could be inferred to partially recover the metabolism pathways of arginine biosynthesis, citrate cycle, and so on. Network pharmacology results suggested that LJP may protect immunosuppressed mice via the regulation of mainly ten biological processes including gluconeogenesis, tricarboxylic acid cycle, and so on. To sum up, our findings suggest that metabolomics method together with network pharmacology would be useful to explore the pathological mechanisms and clarify the mechanisms of the action of LJP for the treatment of immunomodulatory effects.

## CONFLICT OF INTEREST

There are no conflicts of interest to declare.
